# A Provider-Facing eHealth Tool for Transitioning Youth With Special Health Care Needs From Pediatric to Adult Care: Mixed Methods, User-Engaged Usability Study

**DOI:** 10.2196/22915

**Published:** 2021-05-25

**Authors:** Darcy Jones McMaughan, Sherry Lin, Jennifer Ozmetin, Judith Gayle Beverly, Joshua Brog, Emily Naiser

**Affiliations:** 1 Department of Health Policy and Managment School of Public Health Texas A&M University College Station, TX United States; 2 Oklahoma State University Stillwater, OK United States; 3 Public Policy Research Institute Texas A&M University College Station, TX United States

**Keywords:** youth with special health care needs, health care transitions, eHealth, usability, concurrent think aloud method

## Abstract

**Background:**

There is a need for medical education on health care transitions for youth with special health care needs. The Texas Transition Toolkit (the tool) supports providers through a *one-stop shop* for researching literature on care transitions, a catalog of care transition tools, and guides for developing care transition programs.

**Objective:**

This study aims to assess the functionality and usability of the tool with providers working with transition-aged children and youth with special health care needs (representative users).

**Methods:**

The tool was evaluated using a triangulated mixed methods case study approach consisting of a concurrent think-aloud phase, a satisfaction survey, and a survey of problem relevance and task performance to operationalize and capture functionality and usability. Our mixed methods deep dive into the functionality and usability of the tool focused on 10 representative users from one medical home in Texas and 5 website design experts.

**Results:**

Representative users found the tool to be highly relevant, as demonstrated by the satisfaction score for *relevance* (138/150, 92%). According to the users, the tool provided comprehensive information related to health care transitions for youth with special health care needs, with a satisfaction score of 87.3% (131/150) for *comprehensive*. Overall satisfaction with the tool was high at 81.92% (1065/1300) with a cutoff score of 73.33% (953.4/1300) indicating high satisfaction, but users reported relatively lower satisfaction with *search* (114/150, 76%) and *navigation* (*ease of use*: 114/150, 76%; hyperlinks: 163/200, 81.5%; structure: 159/200, 79.5%). They experienced search- and navigation-related problems (total problems detected: 21/31, 68%) and, based on quality checks, had a relatively low task completion rate for tasks involving finding information (60/80, 75%), which required searching and navigation. The problems identified around search and navigation functionality were relevant (relevance scores ranging from 14.5 to 22, with a cutoff score of 11.7 indicating relevance).

**Conclusions:**

The tool may help bridge the gaps in training on health care transitions for youth with special health care needs in US medical education. The tool can be used to create structured protocols to help improve provider knowledge, collaboration across pediatric and adult care providers, and the continuity of care as youth with special health care needs transition from pediatric to adult care. The results provided a road map for optimizing the tool and highlighted the importance of evaluating eHealth technologies with representative users.

## Introduction

### Background

Pediatric to adult health care transitions are a health promotion priority for youth with special health care needs in the United States [1]. Youths with special health care needs have chronic physical, developmental, behavioral, or emotional conditions that require health care and other services beyond typical use [2]. These youths need a higher level of well-coordinated health care, often across multiple providers and specialty types, to realize optimal health and wellness [3,4]. Common conditions among youth with special health care needs include a range of diagnoses that necessitate an even broader range of types and intensities of health care transition support. Diabetes, sickle cell disease, autism, and spina bifida are examples of the diversity of diagnoses among youth with special health care needs. They may need differing care transition support for a variety of aspects of their health care, such as medication management, health status monitoring, physical therapies, and behavioral therapies.

An estimated 5 million youths with special health care needs in the United States are between 12 and 17 years old—the *transition age*, during which health care transition planning should begin [5]. Seamlessly moving from pediatric to adult health care is a critical aspect of transitioning from childhood to adulthood for youth with special health care needs, and structured pediatric to adult care transition programs are associated with positive outcomes [6-8]. Without appropriate health care transition services, youths with special health care needs are vulnerable to adverse outcomes, such as loss of continuity of care [9,10], preventable morbidity, emergency department visits, and hospital visits [11,12].

Pediatric to adult health care transitions should be characterized by having an organizational policy in place that supports care transitions, developing tracking and monitoring processes that follow youths with special health care needs as they transition, assessing youth transition readiness, and transferring care [13]. According to the care transitions consensus statement by the American Academy of Pediatrics, the American Academy of Family Physicians, and the American College of Physicians, providers who care for youth with special health care needs should “(1) understand the rationale for transition from child-oriented to adult-oriented care; (2) have the knowledge and skills to facilitate that process; and (3) know if, how, and when transfer of care is indicated” [14]. It is also vitally important for health care institutions to have organizational policies that support care transitions at the pediatric and adult care levels. The adult team is crucial in supporting care engagement post transfer of care, but much of the emphasis is placed on the pediatric team.

Most youths with special health care needs do not receive adequate care transition services. Across the United States, only 17% of youths with special health care needs received appropriate care transitioning support [15], and access to health services decreased as the youths aged into adulthood [16]. Lack of adequate care transition support may be exacerbated by barriers to care. Although many barriers to care transitions exist for youth with special health care needs, providers also experience barriers to providing care transition services. These barriers can include lack of communication and coordination between the pediatric and adult sides of care, the perceived difficulty of working with youth with special health care needs, and provider hesitation to be involved in the care transition process [17,18]. Provider hesitation may be because of discomfort in providing care transition services, perceived lack of knowledge, lack of training on providing care for youth with special health care needs, and lack of resources (time and low or no reimbursement) associated with providing care transition services [17,19-22].

To assist in addressing issues related to lack of information, communication, and limited resources surrounding care transitions for youth with special health care needs, we tested the functionality and usability of an internet communication technology developed to assist health care providers in transitioning youths from pediatric to adult care. Funded by the Texas Department of State Health Services under the auspices of the federal Maternal and Child Health Bureau, this particular internet communication technology, the Texas Transition Toolkit [23], is an eHealth initiative providing three main services: a *one-stop shop* to research literature on transition care, a catalog of relevant tools to assist providers in creating care transition plans, and steps for how to develop and communicate a transition program. This tool is a state-level initiative that complements the federal Got Transition program [24] and is in line with the Health Resources and Services Administration Maternal and Child Health Bureau Title V Services Block Grant Program’s transition performance measure, specifically advocating for increasing the number of youths receiving appropriate care transition services [25].

Internet communication technologies such as this tool have the potential to provide rapid access to health and medical knowledge. Under one of the many definitions of *eHealth*, these health care system–oriented applications can improve access to quality health care at lower costs. Through eHealth applications, health-related information and knowledge can reach rural, isolated, low-population, or impoverished communities with very little marginal cost to the community or health service provider [26]. As these benefits of eHealth internet communication technology are mitigated by the quality of the application, it is important to evaluate the functionality and usability of eHealth internet communication technology with end users. Functionality assesses whether the technology works the way it is intended to work and delivers the expected results, whereas usability assesses users’ reactions to and interactions with the technology (including perceived utility and satisfaction) [27].

### Objective

This paper describes the formative evaluation of the functionality and usability of this eHealth internet communication tool using a mixed methods usability test. As pragmatic researchers, we determined our research method through our research question: do representative users find the tool functional and usable? [28]. The project was exempted from the institutional review board of the Texas A&M University.

## Methods

### Study Design

The usability test used a triangulated mixed methods case study approach consisting of a concurrent think-aloud phase, a satisfaction survey, and a survey of problem relevance and task performance to operationalize and capture functionality and usability [29,30]. The triangulation mixed methods design uses quantitative and qualitative methods to study an issue [29]. Triangulation considers the qualitative and quantitative aspects of a single study separately during the discovery and analyses stages but integrates the findings of each paradigm to develop new knowledge [31]. This approach examines multiple cases, in this case, multiple representative users, to more fully understand the phenomenon of interest—the functionality and usability of the tool [32]. The evaluation of the functionality and usability of the tool involved assessing for two aspects of the experiences of representative users: a qualitative exploration of problems encountered and a quantitative assessment of satisfaction. To confirm the outcomes of the usability test and further measure the functionality of the tool, the research team explored the relevance of the detected problems and representative users’ task performance. If the number and type of tasks and associated quality checks successfully completed or failed aligns with the concurrent think-aloud and the satisfaction analyses, it supports both the robustness of the results and indicates functionality and usability issues for representative users. Similarly, the presence of *high-relevance* problems in the concurrent think-aloud and satisfaction analyses adds further support to the robustness of the results in detecting important problems and where the functionality and usability of the tool possibly fails. Relevance, in this context, is how connected the type of problem is to the functionality and usability of the tool. It consists of the likelihood of the problem occurring combined with the impact the problem has on the functionality and usability of the tool for representative users, should the problem occur*.*

### Participants

Two groups of participants were recruited for the usability test. One group consisted of representative users—providers working with youths with special health care needs as they transitioned from pediatric to adult care. This group participated in the concurrent think-aloud phase of the usability test and completed the satisfaction survey, thus providing information on problems encountered when using the tool and end user satisfaction with the tool. The second group consisted of website design professionals with experience in designing informational websites for governmental organizations. This group provided information on the relevance of the problems encountered by the representative users.

On the basis of the criterion sampling, 10 representative users were recruited from a medical home in Texas [33]. As the think-aloud method provides a rich source of data, a small sample of subjects generally suffice to discover individual (rather than population) knowledge and experiences [34]. The medical home was an opportunity sample that met the usability trial criterion established a priori, that is, the medical home expressed an interest in a care transition program for youth with special health care needs, had the ability to implement a care transition program, provided care for at least 25 youths with special health care needs, and was able to suggest a champion of care transitions. Through this champion, we were able to recruit a full sample of representative users during a professional conference on care transitions held at the medical home. To be eligible to participate as representative users, the providers must have experience working with youth with special health care needs on the pediatric or adult side of care.

Five website design experts from a research organization affiliated with higher education and a robust history of government website design were recruited to provide expert assessment of the relevance of each problem encountered by representative users during the concurrent think-aloud phase of the usability test. The experts were recruited via snowball recruitment, starting with a government website design consultant familiar with internet communication technology. These experts included software application developers (n=2), web application developers (n=2), and a web and information designer (n=1) with experience in the field ranging from 11 to 17 years.

### Qualitative and Quantitative Assessments

#### Qualitative: Exploring Problems Encountered Through the Concurrent Think-Aloud Method

eHealth internet communication tools can be evaluated using a variety of techniques; most originate from the fields of human-computer interaction and media design, which can be broadly grouped into two modes: expert-focused methods and user-focused methods. One example of a user-focused method is the think-aloud usability testing method [35]. This approach, which originates in the evaluation of physical tasks and then used primarily for software evaluations, is also frequently used for evaluating websites [36,37]. A form of the think-aloud approach, the concurrent think-aloud method, is an evaluation method that involves representative users completing tasks using the tool and simultaneously verbalizing their thoughts. During the recorded sessions, representative users completed tasks according to a predetermined scenario (a vignette) while verbalizing their experiences. Analyses of these verbal reports provided detailed insights into the functionality and usability problems encountered by representative users.

To evaluate the functionality and usability of the tool using the concurrent think-aloud method, the research team developed a set of tasks related to the intended purpose of the tool. For example, representative users were asked to *find a peer-reviewed article focusing on care transitions for youth with type 1 diabetes* and to denote the completion of the task (yes or no). All tasks could be carried out independently to minimize dependency bias and reduce the risk that users would stall after one or two tasks. When applicable, a task contained an associated quality check. The quality check served to determine if the user actually completed the task successfully, in addition to perceived successful completion. These quality checks consisted of asking representative users to write down and speak aloud a few words from the completed subtasks—for example, when asked to find a peer-reviewed article on care transitions for youth with type 1 diabetes, users were subsequently asked to write and speak aloud the first three words of the article title. The tasks were introduced by a vignette, which explained the context and provided the details necessary to perform the tasks. The vignette described a youth with type 1 diabetes poised to graduate high school, transition to attending university, and transition to adult health care without the support of family. Each task represented an action that representative users were likely to perform when using the tool to provide care transition assistance for the youth in the vignette: navigating to pages containing information on care transitions, finding and downloading articles from the evidence base, locating care transition tools, and gathering information on key aspects of developing a care transition program (Multimedia Appendix 1).

During the conference intermission, representative users received concurrent think-aloud tasks and oral instructions on how to carry them out. These instructions, which the facilitator read out loud from a script for consistency, instructed each user to “think aloud while performing your tasks, and pretend that the facilitator is not there. Do not turn to them for assistance. If you fall silent for a while, the facilitator will remind you to keep talking aloud. Finally, remember that it is the T3 Website, and not you, that is being tested.” All interactions between the representative users and the tool were recorded.

#### Quantitative: Assessing Satisfaction With the Website Evaluation Questionnaire

To assess satisfaction with the tool, representative users completed the Website Evaluation Questionnaire upon finishing the concurrent think-aloud phase of the usability test. The Website Evaluation Questionnaire is a 26-item Likert-type scale (Multimedia Appendix 2). Items from the questionnaire load onto one of eight dimensions: ease of use, hyperlinks, structure, relevance, comprehension, completeness, layouts, and search options. For each item, participants circled the response that best characterized their attitude on the item statement: strongly disagree, disagree, neither agree nor disagree, agree, and strongly agree. Each dimension also contained one reverse-coded item. Responses to these items thus represented a disagreement, meaning disagreement with a negative statement about the tool (in other words, satisfaction with the tool along that measure; see Elling et al [35] for the structure of the Website Evaluation Questionnaire).

The Website Evaluation Questionnaire was specifically designed for the evaluation of government websites and is based on user attitudes toward three global factors: the interaction process, the outcomes of the interaction process, and the esthetics of the website [38]. Each of these global factors provided the conceptual foundation for the four main quality factors assessed by the Website Evaluation Questionnaire. The first factor is navigation, which assesses user satisfaction regarding the process of seeking information via the tool. This is particularly relevant to measure the functionality and usability of the tool, given that the purpose of the tool is to assist representative users in finding information on care transitions for youth with special health care needs. Navigation is assessed through the dimensions of ease of use, hyperlinks, and structure, each of which was measured by three (ease of use) or four (hyperlinks and structure) items. The items interrogate users on, for example, whether the website is user friendly (“I consider this website user friendly”), whether they could find the information they needed via the hyperlinks (“under the hyperlinks, I found the information I expected to find there”), and whether the structure of the website supports information-seeking (“the convenient set-up of the website helps me find the information I am looking for”). The second dimension, content, measured the outcomes of the process of seeking information and contained the construct relevance, completeness, and comprehension. Each of these constructs was measured by three items per construct that focused on the perceived utility of the website (ie, “this website offers information that I find useful”), how easy it is to understand the website (“I find the information in this website easy to understand”), and whether the website provides enough information (“this website provides me with sufficient information”). The third dimension was layout, which assesses the esthetics of the website through three items, which focused on the appeal and attractiveness of the website (ie, “I like the way this website looks”). The fourth and final dimension, search options, contained three items assessing the usefulness of information retrieved through the search process (ie, “the search option on this website gives me useful results”). The Website Evaluation Questionnaire was tested in controlled and real-life settings and found to be valid and reliable [35].

#### Quantitative: Assessing the Relevance of Problems Encountered and Task Performance

To assess the relevance of problems encountered with the tool, the web design experts participating in this aspect of the study were asked to evaluate the detected problems in terms of likelihood and impact. Our research team explained to the web design experts that we analyzed recordings of a usability test of the tool and identified verbal indicators of problems experienced by representative users, such as expressions of doubt, task difficulty, incomprehensibility, or annoyance related to the use of the tool. We further explained that to measure the relevance of the problems detected, we asked them, as experts in web design, to rate each problem on a scale of 1 to 5 using two Likert-type scales—one for likelihood and one for impact. Likelihood was defined for the experts as how likely a typical user would experience the problem detected. Impact was defined as how much impact the detected problem would have on the functionality and usability of the website, should that problem occur. We included quotes from the representative users as examples of the detected problems and provided the tool website address for reference (Multimedia Appendix 3).

### Analysis

Once the usability test was completed, the research team transcribed the verbalizations, abstracted data from the surveys, and charted the participants’ navigation through the tool. The functionality and usability of the tool were understood through a thematic analysis of the concurrent think-aloud data using NVivo 12 (QSR International) [39], and descriptive analyses of the Website Evaluation Questionnaire, task performance, and relevance data using Microsoft Excel [40] and Stata 15 (StataCorp) [41].

#### Qualitative: Concurrent Think-Aloud Method

This analysis identified the number and types of problems detected through deep readings of recordings of the usability test sessions. To analyze the data obtained from the concurrent think-aloud aspect of the usability test, the researchers (DJM and JO) used a summative content analysis to identify and quantify the content of the data and understand the contextual use of words and phrases within the content [42]. These words and phrases were synthesized into salient categories (problem types). During this analysis, the researchers interpreted and assigned meanings to the synthesized categories [42]. Problems identified were confirmed through team discussions.

#### Quantitative: Satisfaction With the Tool

Data from the Website Evaluation Questionnaire were coded based on the range of numerical values of each Likert-type item (1-5). Each numerical value in the range weighted the responses to produce a weighted score with *strongly disagree* weighted with 1 and *strongly agree* weighted with 5 and with negative items reverse coded. The weighted score was used to generate a satisfaction ratio based on the sum of the 26 items through a ratio of the total score to the highest possible score, with the highest possible score representing unanimous strong agreement across all representative users. The ratio was then multiplied by 100 to generate the overall satisfaction score. A dimension satisfaction score was similarly generated for each of the eight dimensions.

Cutoff points were determined by subtracting the lowest score on the Likert scale (260) from the highest score on the scale (1300) and dividing by the number of levels of satisfaction (3—for low, medium, and high satisfaction), thus creating an interval value of 346.6. This value was added to each score to create three categories of satisfaction: low (260-606.6), medium (606.7-953.3), and high (953.4-1300). By applying these cutoff scores to the ratio of the total score to the highest possible score, we determined that a satisfaction score of 73.33% (953.4/1300) is the cutoff for high satisfaction. The same process was repeated across dimensions for satisfaction cutoff scores of low (30-70), medium (71-111), and high (112-150) for three item dimensions and low (40-93.3), medium (93.4-146.7), and high (146.8-200) for four item dimensions. The resulting high satisfaction cutoff score was 74.6% (112/150) for the three item dimensions and 73.4% (146.8/200) for the four item dimensions. Sensitivity analyses were conducted using cutoff scores based on mean and mode with no changes to the results.

#### Quantitative: Relevance of the Problems Encountered and Task Performance

The relevance score for each problem was created through the square root of the multiplied likelihood and impact scores [43]. Likelihood and impact scores were created by weighting each numerical value in the range of the responses to produce a weighted score with *unlikely* or *no impact* weighted with 1 and *highly likely* or *high impact* weighted with 5. The weighted score was used to generate a likelihood or impact score for each problem through a ratio of the total score to the highest possible score, with the highest possible score being 25. Cutoff points were determined by creating a three-level (low, medium, and high) interval value. A score of 5 to 11.6 indicated low likelihood, low impact, or low relevance. A score of 11.7 to 18.3 was indicative of likely, impactful, and relevant. A score of 18.4 to 25 signified that the problem type was highly likely, had high impact, or was highly relevant. Task performance was understood through the completion rate for each task and the associated quality checks.

## Results

### Representative Users

Given the small sample size and potential for identification, demographic data of representative users were fuzzed. Approximately 90% of the representative users who participated in the usability test were White, non-Hispanic women. Every user worked with at least one youth with special health care needs, and approximately 90% had a professional caseload where more than half of the patients were youths with special health care needs. More than one-third of the users worked solely with youths with special health care needs. Almost all (approximately 80%) of the representative users transitioned youths from pediatric to adult care. Half of the users worked with youths with special health care needs on both ends of the continuum—pediatrics and adult care. Approximately one-fourth of the users were physicians and approximately another quarter were nurse practitioners. The remaining representative users were social workers (approximately one-fourth), nurses, and transition specialists (clinical team coordinators, youth service specialists, and transition coaches). Some users served multiple roles (eg, nurses and certified educators for a particular chronic condition).

### Qualitative: Problems Encountered Through the Concurrent Think-Aloud Method

Table 1 displays the number of problems detected, the different types of problems detected, and the frequency of each type of problem. A total of 31 problems were detected through the analysis of the concurrent think-aloud transcripts. These problems were aggregated into 10 categories or type of problem, most of which (29/31, 93%) focused on issues associated with finding information on health care transitions, such as the utility of search criteria, finding the search bar, finding disease-specific resources, and finding resources blocked by a firewire. Among these problems, approximately one-fourth (8/31, 26%) centered on the utility of search criteria, meaning representative users found it difficult to create search terms that would return resources on a particular topic in health care transitions:

Still looking...self-care management, maybe I’m looking in the wrong place. Care transition, EPIC transition planning tool...uh I’m not finding the self- care. Okay, I’ll probably give up on that.

**Table 1:** Frequency of detected T3 problems by type (N=31).

Problem type	Examples	Frequency, n (%)
Utility of search criteria	“Okay so if I type in the wrong thing it makes it more difficult. Still looking...self-care management, maybe I’m looking in the wrong place. Care transition, EPIC transition planning tool...uh I’m not finding the self- care. Okay, I’ll probably give up on that.”	8 (26)
Finding the search bar is difficult	“I’m looking for that, scrolling. Hmm, is there a search bar? That would have been easy, oh here we go, I found it.”	4 (13)
Finding disease-specific resources	“So, I still think that it would be better to organize this page by general versus...and then also you could have some general articles and then you could have some disease-specific articles and the disease-specific articles could be in alphabetical order to make it easier to find because I kinda gave up on that one.”	3 (10)
Email of website contact opens email software or application	“Oh okay, so you have to add an account, so you have to actually put your email in? Okay can I close that?”	3 (10)
Cannot locate the email of the website contact person	“Copy any contact email address. Let’s see. Okay so I’m going to the wrong place and I’m going back to look. Okay, so it has a contact person but no email address.”	3 (10)
Need clarity on who to contact for an article behind a firewall	“I can’t really find who to talk to about getting this article.”	3 (10)
Some windows blocked by a firewall or files won’t open	“There were, I’m going to score it a four because there were some windows blocked by a firewall.”	3 (10)
Search bar is missing	“Is there a search bar here? That would be helpful under the tools, search for articles and tools.”	2 (6)
Clicking on aspects of the webpage results in no action	“I should not be doing this because when I click on it, it doesn’t work.”	1 (3)
Difficulty returning to a previous page (*going back*)	“Do I hit back-arrow or close?”	1 (3)

In total, 19% (6/31) of the problems resulted from the difficulty in finding the search bar. The representative users found the placement of the search bar confusing to the point that some thought the search bar was missing:

Is there a search bar here? That would be helpful under the tools, search for articles and tools.

Approximately 10% (3/31) of the problems were associated with users encountering difficulties finding disease-specific resources:

So, I still think that it would be better to organize this page by general versus...and then also you could have some general articles and then you could have some disease-specific articles and the disease- specific articles could be in alphabetical order to make it easier to find because I kinda gave up on that one.

When attempting to access resources behind a firewall, users encountered a number of problems, including blocked windows, software application openings, and an inability to access the contact information of the website manager:

I can’t really find who to talk to about getting this article.

### Quantitative: Satisfaction With the Tool

The overall satisfaction score for the tool was 81.92% (1065/1300), indicating high satisfaction based on the overall satisfaction cutoff score of 73.33% (953.4/1300). The tool scored highest in relevance (138/150, 92%), followed by comprehension and layout (both 131/150, 87.3%), and the lowest in search options (114/150, 76%) and ease of use (114/150, 76%; Table 2 [35]). Satisfaction was high for each dimension.

**Table 2:** Representative user satisfaction with the tool based on the Website Evaluation Questionnaire.

Dimension and item^a^			Satisfaction scores		
			Dimension score, n (%)		Item score, n (%)
**Relevance**			138 (92)		
	I find the information in website helpful.			46 (92)	
	Website offers information I find useful.			47 (94)	
	Information in this website is of little use to me.^b^			45 (90)	
**Comprehension**			131 (87.3)		
	Language used in website is clear to me.			46 (92)	
	I find the information in website easy to understand.			42 (84)	
	I find many words in website difficult to understand.^b^			43 (86)	
**Layout**			131 (87.3)		
	I like the way this website look.			42 (84)	
	I find the design of this website appealing.			43 (86)	
	I think this website looks unattractive.^b^			46 (92)	
**Structure**			159 (79.5)		
	I know where to find information I need on this website.			38 (76)	
	I find the structure of this website clear.			42 (84)	
	I was constantly redirected on this website.^b^			39 (78)	
	The convenient set-up of the website helps me find the information I am looking for			40 (80)	
**Hyperlinks**			163 (81.5)		
	Homepage clearly directs me towards information I need.			39 (78)	
	Homepage immediately points me to information I need.			40 (80)	
	Under hyperlinks, I found information I expected to find.			43 (86)	
	It is unclear which hyperlink leads to information I need.^b^			41 (82)	
**Completeness**			115 (76.6)		
	This website provides me with sufficient information.			41 (82)	
	I find the information in this website precise.			37 (74)	
	I find the information in this website incomplete.^b^			37 (74)	
**Search options**			114 (76)		
	Search option helps me find the right information quickly.			38 (76)	
	Search option gives me useful results.			40 (80)	
	Search option gives me too many irrelevant results.^b^			36 (72)	
**Ease of use**			114 (76)		
	I find this website easy to use.			38 (76)	
	I consider this website user friendly.			38 (76)	
	I had difficulty using this website.^b^			38 (76)	

^a^Item wording truncated for parsimony. Please see the Website Evaluation Questionnaire for complete item wording.

^b^Reverse-coded items. The score represents a disagreement score, meaning disagreement with a negative statement about the tool (thus satisfaction with the tool along that measure).

### Quantitative: Relevance of the Problems Encountered and Task Performance

Table 3 shows task performance. This is the number of tasks and quality checks successfully completed. Overall, 89% (89/100) of all the tasks and 75% (60/80) of the quality checks were completed successfully. Participants were least likely to find a specific tool for early adolescence, with a 70% (7/10) completion and a 70% (7/10) quality check success rate.

**Table 3:** Participant (n=10) task performance and associated quality checks.^a^

Task and check^b^			Completion, n (%)
**Navigate to page contains a database of peer-reviewed articles.**			9 (90)
	Write the first 3 words of the database page title.	8 (80)	
**Find peer-reviewed article on care transition for T1 diabetes.**			10 (100)
	Write the first 3 words of the article title.	8 (80)	
Download the article and view the abstract.			10 (100)
Navigate back to the homepage.			9 (90)
**Find the contact information of someone from the T3.**			8 (80)
	Copy the contact’s email here.	5 (50)	
**A tool by Parent to Parent provides a timeline and action items for parents of youth with special health care needs. Find and view this tool.**			9 (90)
	Write the first 3 words of the tool title.	7 (70)	
**Find a tool to discuss level of distress around chronic disease self- management.**			9 (90)
	Write the first 3 words of the tool title.	9 (90)	
**You are charged with improving care transition for youth with special health care needs for your institution. Find the page that would be the most useful.**			9 (90)
	Write the page you found this on.	8 (80)	
**According to the T3, is a champion important?**			9 (90)
	Write the page you found this on.	8 (80)	
**Find a tool specific for early adolescence.**			7 (70)
	Write the first 3 words of the tool title.	7 (70)	

^a^Aggregating by tasks and checks, we found an 89% (89/100) and 75% (60/80) completion rate, respectively.

^b^Item wording truncated for parsimony.

Table 4 provides the likelihood, impact, and relevance scores of each problem type. Focusing on relevance, every problem type was found to be relevant based on a cutoff score of 11.7 for relevance, and 60% (6/10) of the problems were found to be highly relevant to the functioning of the tool based on a cutoff score of 18.4 for highly relevant. Problems that interrupted the ability of end users to locate information because of the layout or mechanical workings of the tool were rated as having the highest relevance to the functioning of the tool. These involved the tags programmed on search terms *(trouble finding search criteria that result in what user wants*: relevance=18.4), organization of information (hard to find articles by disease: relevance=21.5), and inability to access information *(email of website contact opens email software*: relevance=20.4; *need clarity on who to contact for article behind a firewall*: relevance=21; *some windows blocked by a firewall or files will not open*: relevance=22).

**Table 4:** Relevance of each problem type (n=10).

Problem type	Relevance^a^= 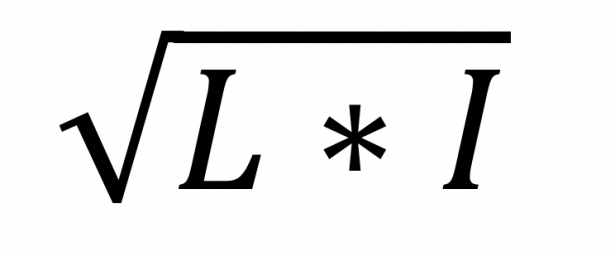 , weighted scores		
	Likelihood	Impact	Relevance
Trouble finding search criteria result in what user wants	20	17	18.4
Finding the search bar is difficult	18	17	17.5
Hard to find articles by disease	21	22	21.5
Email of website contact opens email software or application	19	22	20.4
Cannot locate the email of the website contact person	17	17	17
Need clarity on who to contact for article behind a firewall	20	22	21
Some windows blocked by a firewall or files won’t open	22	22	22
Search bar is missing	20	24	21.9
Clicking on aspects of the webpage results in no action	14	18	15.9
Difficulty returning to a previous page (“going back”)	15	14	14.5

^a^Relevance=
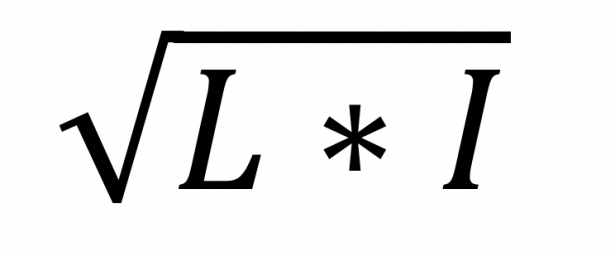
 or the square root of the likelihood score×the impact score (Van den Haak et al [43]). Cutoff scores were 5-11.66 for low relevance, 11.67-18.33 for relevant, and 18.34-25 for highly relevant.

## Discussion

### Principal Findings

The functionality and usability testing of the tool with representative users highlighted the usefulness of this eHealth internet communication technology among providers working with youth with special health care needs. Representative users found the tool to be highly relevant, as demonstrated by the satisfaction score for the dimension *relevance* (138/150, 92.0%). According to the users, the tool provided comprehensive information related to health care transitions for youth with special health care needs, with a satisfaction score of 87.3% (131/150) on the comprehensive dimension. This is important, as it suggests that the tool may help bridge the gaps in training on health care transitions for youth with special health care needs in US medical education [44]. Gaps in education on health care transitions exist at all levels of medical education [44], and providers often express concerns about the lack of knowledge on transitioning care for youth with special health care needs [45,46]. Furthermore, youths transitioning from pediatric to adult care also want access to medical education on health care transitions [47], which can be provided through the tool. There is also a need to provide information on health care transitions among youth with special health care needs to adult providers, who may lack knowledge of the process [48]. An eHealth internet communication technology such as the tool, which includes evidence-based literature on health care transitions and templates for transition protocols, can be used to create a structured tool to help improve collaboration across pediatric and adult care providers and continuity of care [49]. The tool does not rely on the ability of a health care organization to integrate the tool into their system; it can be implemented within an organization or external to an organization. Although integration within the systems of care is ideal [50], in other studies of educational transition tools, health care providers expressed concern over the ability to integrate tools, given the characteristics and deficits of health care systems [51].

Although the tool was well received by representative users, the usability test results identified areas of concern regarding functionality and usability. Users reported the most difficulty in two areas of functionality and usability: search and navigation. This was reflected in both the concurrent think-aloud and satisfaction survey results and was supported by the task performance and relevance analyses. Representative users reported lower satisfaction with search and navigation dimensions, relatively high number of search- and navigation-related problems and low task completion for tasks involving finding tools that require searching and navigation. The problems identified around the search and navigation functionality were also found to be relevant by web design experts. Each of these areas of analyses triangulates on search and navigation issues, suggesting the robustness of the results and allowing researchers to fine-tune the tool to optimize performance.

Uncovering these inefficiencies and subsequently optimizing the tool improves its functionality and usability, which may drive the future use of the technology for its specified purpose [52]. Studies of commercial technologies show that end users are more likely to utilize a technology if it is easy to use [53-55]. This is especially important for eHealth internet communication technology, such as the tool, which is intended to improve health outcomes in the long term through the dissemination of information and best practices. Although the results from the usability test provide a clear road map for optimizing the tool, they also highlight the importance of evaluating eHealth and internet communication technology with representative users. Often, internet communication technology evaluations focus too intensely on the technical aspects of the technology rather than the needs and expectations of end users [56]. Conducting functionality and usability assessments before widespread implementation is essential for forecasting usage and ensuring that internet communication technology has the intended impact [57].

### Limitations and Future Research

Although the concurrent think-aloud methodology and the satisfaction survey provided a robust way to ascertain the functionality and usability of this eHealth internet communication technology, there are certainly other acceptable methods. In fact, even with the growing interest in website functionality and usability and the growing use of internet communication technology for eHealth applications, there is no consensus on the definition of usability. There exists a multitude of possible dimensions and measurement techniques in the field of internet communication technology research. Choosing between these dimensions and techniques to evaluate the technology involves a certain amount of bias, which could result in the website performing better in the laboratory than in a real-life setting. However, this usability test followed quality measures for usability studies of eHealth applications [58]. We used a valid and reliable tool, chose our study design based on the objectives of the study, used across-method triangulation, and included both representative users and experts in assessing usability.

Few evaluated measures exist that can be used to understand the impact of eHealth technology on health outcomes [59], which has potentially contributed to the limited evaluation of eHealth [60]. This is a particularly important limitation when the website is eHealth internet communication technology and is expected to affect health outcomes. Thus, further research is needed on the effect of the tool on care transition outcomes, particularly in isolated or resource-poor communities. Evaluation research on holistic care transition tools and programs is limited, with the majority of outcomes-focused evaluations targeting narrow, disease-specific populations of youth with special health care needs [6]. Although disease-specific instruments fall under the triple aim domain of population health [61], considering that most youths with special health care needs experience multiple comorbid conditions across their lifetime, more empirical evidence is needed on tools dedicated to broader care transitions. This tool, with its broad focus, answers calls to assist care providers in transitioning youth with special health care needs using a biopsychosocial model [62].

## Appendix

Multimedia Appendix 1 Texas Transition Toolkit think-aloud usability testing: vignette, tasks, and quality checks.

Multimedia Appendix 2 The website evaluation questionnaire.

Multimedia Appendix 3 Texas Transition Toolkit usability trial: website problem relevance.
