# Supplementary material for: A Provider-Facing eHealth Tool for Transitioning Youth With Special Health Care Needs From Pediatric to Adult Care: Mixed Methods, User-Engaged Usability Study
Source: JMIR Form Res. 2021 May 25;5(5):e22915. doi: 10.2196/22915 (PMC8188313; doi:10.2196/22915)
Supplement: Multimedia Appendix 1 [file formative_v5i5e22915_app1.docx]

**Texas Transitions Toolkit (T3) Think Aloud Usability Testing**

Please use the following vignette and tasks to navigate the T3 and answer the questions below. Please think aloud while performing your tasks, and pretend that the facilitator is not there. Do not turn to them for assistance. If you fall silent for a while, the facilitator will remind you to keep talking aloud. Finally, remember that it is the T3 Website, and not you, that is being tested.

T3 Website: http://dshs.wpengine.com/

**Charlie is 16 and has Type 1 diabetes**

Charlie is an 16-year old student who is about to graduate high school with honors and is looking forward to attending university soon. Charlie has Type 1 Diabetes. Charlie has seen a pediatric endocrinologist in their hometown for several years. Charlie’s parents are highly involved in caring for their child. This includes regulating Charlie’s blood glucose levels. Sometimes Charlie’s parents checks on them while they are sleeping to ensure Charlie’s blood glucose is at a safe level. With Charlie gaining a new level of independence by possibly moving to the university, Charlie must also take on additional responsibility in controlling their blood glucose. Charlie has always been able to rely on their parents when they need help, but moving to university means Charlie will be solely responsible in the next few years.

**TASKS**

1. **Navigate to a page on the T3 that contains a database of peer-reviewed articles**.

Completed? Yes

 No *

*

Page Title (first 3 words):

1. **Find a peer-reviewed article focusing on care transitions for youth with Type 1 Diabetes.**

Completed? Yes

 No *

*

Article Title (first 3 words):

1. **Download the article and view the abstract.**

Completed? Yes

 No *

*

1. **Navigate back to the T3 Homepage.**

Completed? Yes

 No *

*

1. **You found an article in the database that you’d really like to read, but it’s behind a firewall. Find the contact information of someone who can help you from the T3.**

Completed? Yes

 No *

*

Copy the contact’s email here:

1. **Charlie’s parents are concerned about what they can do to help Charlie. There is a tool by Parent to Parent that provides a timeline and suggestions for discussions and action items for parents of CYSHCN. Find and view this tool.**

Completed? Yes

 No *

*

Tool Title (first 3 words):

1. **Charlie mentioned struggling with the burden of diabetes self care and management. Find a tool that would help you and Charlie discuss their level of distress around chronic disease self management.**

Completed? Yes

 No *

*

Tool Title (first three words):

1. **You are charged with improving care transitions for CYSHCN for your institution. Find the T3 page that would be the most useful.**

Completed? Yes

 No *

*

Page Title (first 3 words):

1. **According to the T3, is a champion important?**

Completed? Yes

 No *

*

Page Title (first 3 words):

1. **Find a tool specific for early adolescence.**

Completed? Yes

 No *

*

Title of Tool (first 3 words):

**Thank you for participating!**
