# Supplementary material for: A Provider-Facing eHealth Tool for Transitioning Youth With Special Health Care Needs From Pediatric to Adult Care: Mixed Methods, User-Engaged Usability Study
Source: JMIR Form Res. 2021 May 25;5(5):e22915. doi: 10.2196/22915 (PMC8188313; doi:10.2196/22915)
Supplement: Multimedia Appendix 2 [file formative_v5i5e22915_app2.docx]

**Form Number:**

**The Website Evaluation Questionnaire (WEQ)**

This questionnaire focuses on the Texas Transitions Toolkit (T3). For each of the questions below, circle the response that best characterizes how you feel about the statement, where:

***1=Strongly Disagree, 2=Disagree, 3=Neither Agree nor Disagree, 4=Agree, and***

***5=Strongly Agree***

**Q1. I find the information on this website helpful.**

1 2 3 4 5

**Q2. The language used in this website is clear to me.**

1 2 3 4 5

**Q3. This website provides me with sufficient information.**

1 2 3 4 5

**Q4. I find this website easy to use.**

1 2 3 4 5

**Q5. The homepage clearly directs me towards the information I need.**

1 2 3 4 5

**Q6. I know where to find the information I need on this website.**

1 2 3 4 5

**Q7. I think this website looks unattractive.**

1 2 3 4 5

**Q8. The information in this website is of little use to me.**

1 2 3 4 5

**Q9. I find the information in this website easy to understand.**

1 2 3 4 5

**Q10. I find the information in this website incomplete.**

1 2 3 4 5

**Q11. I had difficulty using this website.**

1 2 3 4 5

**Q12. The homepage immediately points me to the information I need.**

1 2 3 4 5

**Q13. I was constantly being redirected on this website while I was looking for information.**

1 2 3 4 5

**Q14. I like the way this website looks.**

1 2 3 4 5

**Q15. This website offers information that I find useful.**

1 2 3 4 5

**Q16. I find many words in this website difficult to understand.**

1 2 3 4 5

**Q17. I find the information on this website precise.**

1 2 3 4 5

**Q18. This website provides me with sufficient information.**

1 2 3 4 5

**Q19. It is unclear which hyperlink will lead to the information I am looking for.**

1 2 3 4 5

**Q20. I find the structure of this website clear.**

1 2 3 4 5

**Q21. I find the design of this website appealing.**

1 2 3 4 5

**Q22. Under the hyperlinks, I found the information I expected to find there.**

1 2 3 4 5

**Q23. The convenient set-up of the website helps me find the information I am looking for.**

1 2 3 4 5

**Q24. The search option on this website helps me to find the right information quickly.**

1 2 3 4 5

**Q25. The search option on this website gives me useful results.**

1 2 3 4 5

**Q26. The search option on this website gives me too many irrelevant result**s.

1 2 3 4 5

**Additional Comments:**

Elling, S., Lentz, L., de Jong, M., Van den Bergh, H. (2012). Measuring the quality of governmental websites in a controlled versus an online setting with the ‘Website Evaluation Questionnaire’. Government Information Quarterly, 29(3), 383-393.

Elling, S., Lentz, L., & De Jong, M. (2007, September). Website evaluation questionnaire: development of a research-based tool for evaluating informational websites. In *International Conference on Electronic Government* (pp. 293-304). Springer, Berlin, Heidelberg.
