# Supplementary material for: A Provider-Facing eHealth Tool for Transitioning Youth With Special Health Care Needs From Pediatric to Adult Care: Mixed Methods, User-Engaged Usability Study
Source: JMIR Form Res. 2021 May 25;5(5):e22915. doi: 10.2196/22915 (PMC8188313; doi:10.2196/22915)
Supplement: Multimedia Appendix 3 [file formative_v5i5e22915_app3.docx]

Texas Transition Toolkit (T3) Usability Trial

Website Problem Relevance

Our research team analyzed recordings of a usability trial of the Texas Transition Toolkit website. We examined the transcripts and identified verbal indicators of problems experienced among the study participants, such as expressions of doubt, task difficulty, incomprehensibility, or annoyance related to the use of the T3. To measure the relevance of the problems detected, the research team is asking you, as an expert in web design, to evaluate the detected problems in terms of likelihood and impact.

Please rate the problems below on a scale of 1 to 5 for likelihood and impact. Likelihood is how likely a typical user would experience the problem detected. Impact is how much impact the detected problem would have on the usability of the website, should that problem occur.

Likelihood:1= unlikely and 5=highly likely

Impact: 1=no impact and 5=high impact

We included quotes from the participants (in italics) as examples of the detected problem.

For reference, the website is: <https://www.texastransitiontoolkit.org/> Please note that the current website may be different than what the usability trial participants viewed.

1. **Finding the search bar is difficult**

*Oh, and I’m looking for that, scrolling… Hmm, is there a search bar? That would have been easy, oh here we go, I found it. At first, I didn’t only because of I.T. incompetency***.**

*I’m gonna look under tools, I guess I should’ve used the search bar, I just saw it.*

*Yeah…so there should kind of be a search on this. Oh, it is up here. Never mind, it’s on top.*

*Okay so I discovered the search feature, that was helpful.*

Likelihood

1 2 3 4 5

Impact

1 2 3 4 5

1. **Search bar is missing**

*Is there a search bar here? That would be helpful under the tools, search for articles and tools, yes okay.*

Likelihood

1 2 3 4 5

Impact

1 2 3 4 5

1. **Trouble finding adequate search criteria that results in what the user wants**

*Okay so if I type in the wrong thing it makes it more difficult*

*Still looking…self-care management, maybe I’m looking in the wrong place. Care transitions, EPIC transition planning tool…uh I’m not finding the self-care. Okay, I’ll probably give up on that*

*I didn’t find that one very easily so I’m gonna move on to the next question*

*I’m still reading all these titles so it’s going to take me a minute, evidently, I did not use adequate search criteria.*

*I’m doing all sorts of searches on this thing but I’m not getting what I want.*

Likelihood

1 2 3 4 5

Impact

1 2 3 4 5

1. **Hard to find articles by disease**

*So, I still think that it would be better to organize this page by general versus… and then also you could have some general articles and then you could have some disease-specific articles and the disease-specific articles could be in alphabetical order to make it easier to find because I kinda gave up on that one.*

*I wonder if it would be nice to have each disease listed and the article that corresponds because it’s a little bit hard to have to go all the way down the page to try to find diabetes. I’m still looking for diabetes, maybe I missed it somewhere.*

Likelihood

1 2 3 4 5

Impact

1 2 3 4 5

1. **Email of contact opens Microsoft Outlook application**

*So, do you have to add an account to get the email?*

*Oh okay, so you have to add an account, so you have to actually put your email in? Okay can I close that?*

*I have her number but I did not see an email. Let’s scroll up. I am guessing if I click on her I might be able to…and cool, I clicked on her and it opened email automatically and it’s trying to add an account so we are not gonna do that.*

Likelihood

1 2 3 4 5

Impact

1 2 3 4 5

1. **Can't find email of contact person**

*Copy any contact email address. Let’s see. Okay so I’m going to the wrong place and I’m going back to look. Okay, so it has a contact person but no email address.*

Likelihood

1 2 3 4 5

Impact

1 2 3 4 5

1. **Need more clarity on who to contact if you want an article behind a firewall**

*I can’t really find who to talk to about getting this article.*

*How do you find help getting something past a firewall? I don’t know the answer to that.*

Likelihood

1 2 3 4 5

Impact

1 2 3 4 5

1. **Some windows blocked by a firewall/files won’t open**

*There were, I'm going to give it a four because there were some windows blocked by a firewall.*

*It might open. Nope, it will not open.*

Likelihood

1 2 3 4 5

Impact

1 2 3 4 5

1. **Clicking on aspects of the webpage results in no action**

*I should not be doing this because when I click on it, it doesn’t work because I’m used to the Apple.*

Likelihood

1 2 3 4 5

Impact

1 2 3 4 5

1. **How to ‘go back’**

*Do I hit back-arrow or close?*

Likelihood

1 2 3 4 5

Impact

1 2 3 4 5

THANK YOU!!
